# A Common Symptom With an Uncommon Diagnosis: A Case of Primary Esophageal Diffuse Large B-cell Lymphoma

**DOI:** 10.7759/cureus.51885

**Published:** 2024-01-08

**Authors:** Shruthi Narasimha, Rasiq Zackria, Jonathan Hughes, Vijay Jayaraman

**Affiliations:** 1 Gastroenterology and Hepatology, Sunrise Health Graduate Medical Education (GME) Consortium, Las Vegas, USA; 2 Pathology, LMC Pathology, Las Vegas, USA; 3 Gastroenterology, Comprehensive Digestive Institute of Nevada, Las Vegas, USA

**Keywords:** diffuse large b-cell lymphoma, endoscopy, dysphagia, cancer, esophagus

## Abstract

Diffuse large B-cell lymphoma (DLBCL) is the most common type of non-Hodgkin’s lymphoma. Although it can have gastrointestinal involvement, there are limited recorded cases that show primary esophageal DLBCL. This report discusses the case of an 85-year-old female who initially presented with weight loss associated with dysphagia and was later diagnosed with an esophageal mass by endoscopy. Pathology showed large, atypical lymphocytes, and the final morphologic, immunohistochemical, and molecular findings were most consistent with a diagnosis of primary esophageal DLBCL.

## Introduction

Non-hodgkin’s lymphoma is the most common form of lymphoma, with diffuse large B-cell lymphoma (DLBCL) being the most prevalent [1]. Primary extranodal involvement is noted in up to 50% of patients with DLBCL; the gastrointestinal (GI) tract is the most frequent location for secondary extranodal involvement [2,3]. More than 80% of the DLBCLs in the GI system are found in either the stomach and/or small intestine. Most instances of esophageal involvement typically result as an extension of either mediastinal or gastric involvement; however, the esophagus is a rare primary site and accounts for fewer than 1% of all DLBCL cases [4]. We present a case report of new-onset dysphagia that was subsequently diagnosed with primary esophageal aggressive large B-cell lymphoma.

## Case presentation

An 85-year-old Caucasian woman with a past medical history of Parkinson’s disease, hypothyroidism, and type 2 diabetes mellitus was initially referred to the advanced gastroenterology clinic for evaluation of worsening dysphagia with associated regurgitation for the past two months. Her symptoms were primarily associated with solid food intake and she reported an unintentional 20-lb weight loss that she attributed to poor oral intake. She denied a family history of malignancies. Although she denied current alcohol, tobacco, or recreational drug use, she reported being a 20-pack-year former cigarette smoker. The patient reported her functional status to have worsened over the past few months and now requiring assistance with activities of daily living due to weakness. Due to her worsening dysphagia, she underwent an esophagram that demonstrated a short segment of mucosal irregularity in the mid-esophagus, a small hiatal hernia, and reflux of contrast in the esophagus. She subsequently underwent an esophagogastroduodenoscopy (EGD) that showed an ulcerated hard mass about 25 cm from the incisors. The gastroscope was unable to completely pass the stricture and multiple cold forceps biopsies showed necroinflammatory debris and fibrinopurulent exudate consistent with an ulcer bed with inflamed granulation tissue.

**Figure 1:**
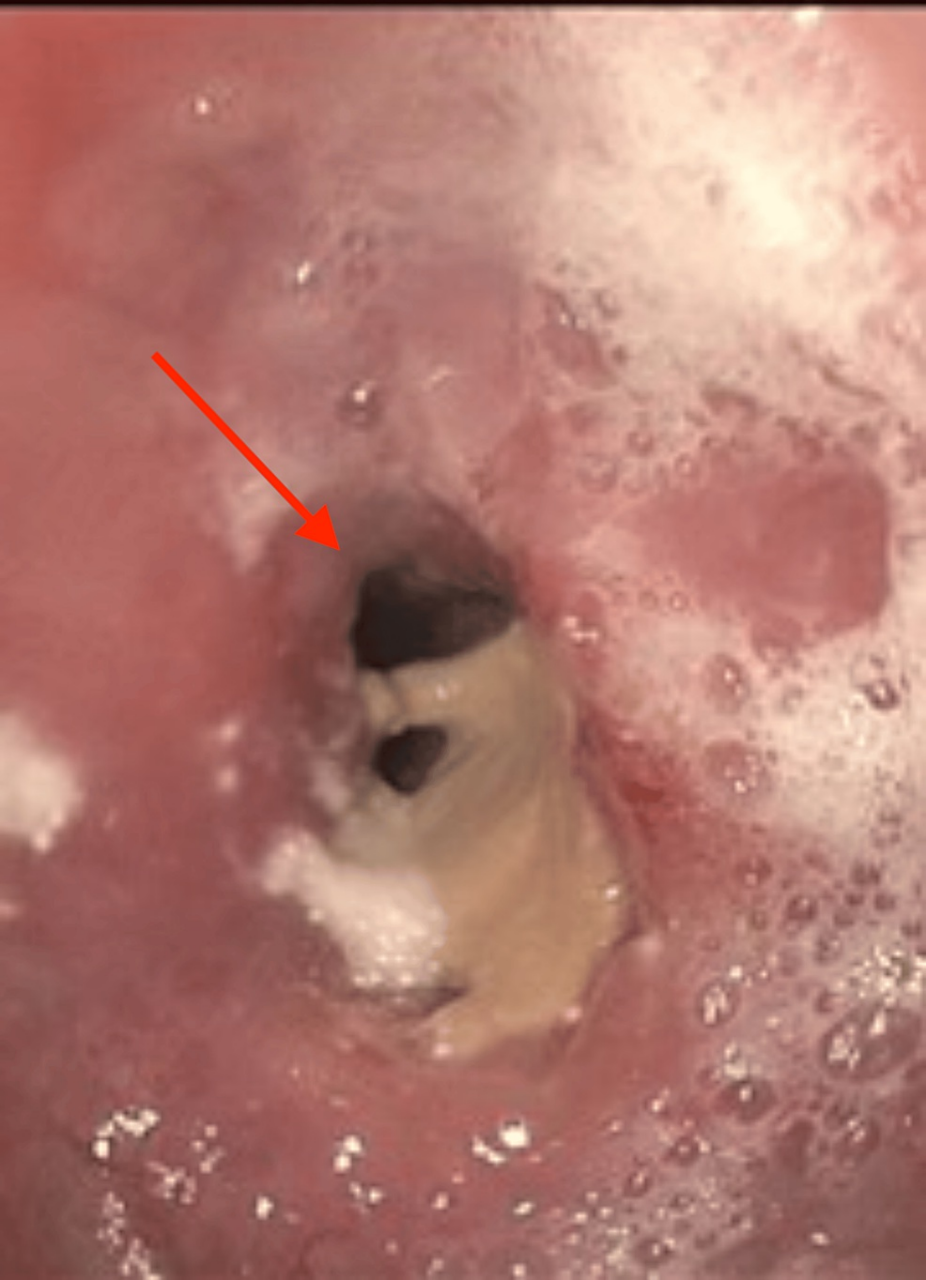
Esophagogastroduodenoscopy showing esophageal ulcerated stenosis (demonstrated by the red arrow) in the mid-esophagus.

**Figure 2:**
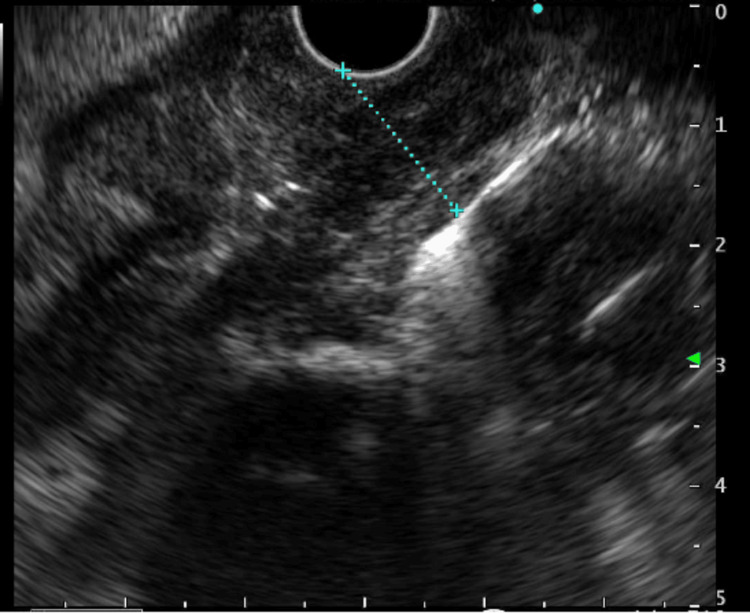
Endoscopic ultrasound depicting a heterogenous mass, as measured by the blue line, is seen invading past the muscularis propria.

Due to high concern for malignancy, the patient was referred for an endoscopic ultrasound (EUS) with fine-needle biopsy. Repeat EGD demonstrated a large, circumferential ulcerated mass at 25 cm from the incisors with luminal stenosis (Figure 1). With some difficulty, the gastroscope was able to bypass the stricture, and the circumferential lesion was measured to be about 7 cm in length. EUS showed a hypoechoic, heterogenous mass invading past the muscularis propria with two affected regional lymph nodes (Figure 2). This led to the staging of this possible malignancy as T3N1. Preliminary pathologic analysis suggested lymphoproliferative cells.

**Figure 3:**
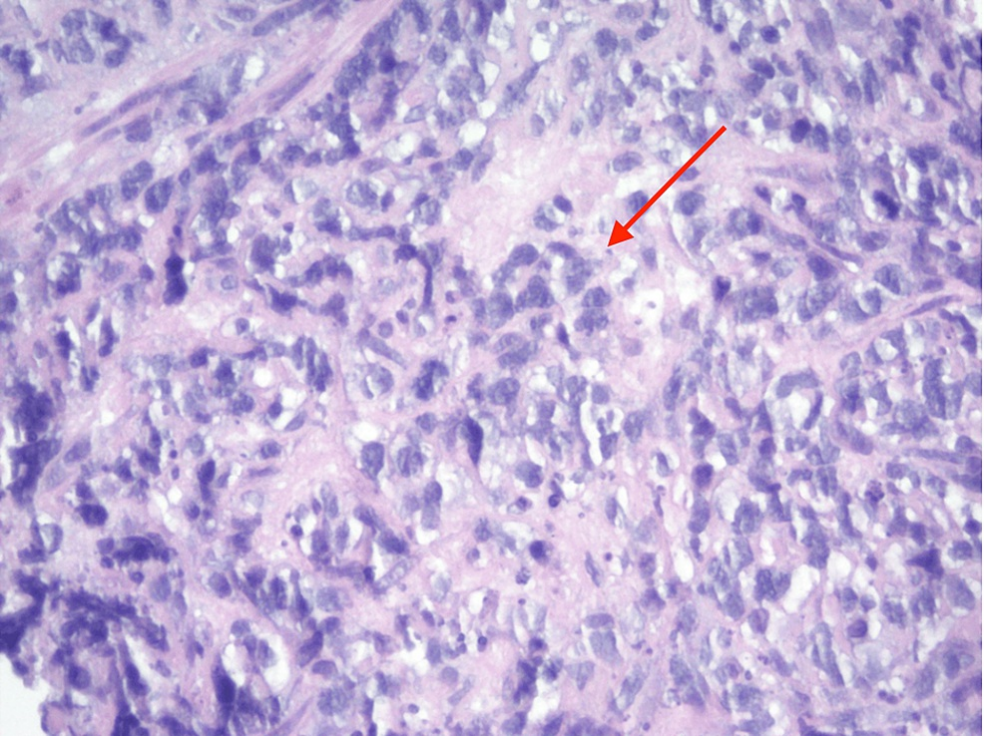
Histopathology with hematoxylin and eosin staining shows large atypical lymphocytes with large nuclei within a dense fibrous tissue background (demonstrated by the red arrow). Micrograph magnified at 200×.

Microscopic examination of the aspirate showed numerous atypical lymphocytes, many of which were crushed in a background of dense fibrous tissue (Figure 3). Following the review of routine stains, a panel of immunohistochemical stains was performed to further classify the lymphoid population. The large, atypical lymphocytes showed positive staining for CD20, BCL2, BCL6, and CD10. A stain for the proliferation marker Ki67 revealed a nuclear labeling index of approximately 100%. The following stains were negative: CD3, pan-cytokeratin, P63, CDX2, cytokeratin 5/6, P16, BCL1, and CD30. Molecular studies demonstrated gains of chromosomes 8 and 18q. Collectively, the morphologic, immunohistochemical, and molecular findings were most consistent with a final classification of DLBCL. There was no evidence of double/triple hit lymphoma.

Unfortunately, the patient was considered to have advanced disease and she decided to pursue hospice care.

## Discussion

The most common esophageal malignancies often arise from epithelial cells and usually turn into squamous cell cancer or adenocarcinoma. Primary esophageal DLBCL, a variant of non-Hodgkin’s lymphoma, is an extremely rare clinicopathologic entity that was initially reported in 1979 [5]. It accounts for fewer than 1% of primary GI lymphomas [6]. It predominantly involves immunocompromised patients, and positive HIV status is a potential risk factor [7]. Fewer than 30 cases of primary esophageal lymphoma have been reported in the literature, and a recent literature review found only 14 cases of confirmed primary esophageal lymphoma without any metastatic disease [8,9].

Initially, Dawson’s criteria was used to diagnose primary esophageal lymphoma. The criteria included mainly involvement of the esophagus, lymphadenopathy limited to only local lymph nodes, no involvement of the spleen or liver, and a normal granulocyte cell value [10]. However, a recent study in 2021 showed that over 40% of patients with the diagnosis did not meet these specifications [8]. Detection of this malignancy is hard due to the lack of specific symptoms as most patients have vague and varied symptoms with dysphagia being the most common. Additionally, gaining diagnostic pathology is made difficult because the tissue needed usually lies deep in the mucosa, an area that regular endoscopic biopsies may not be able to reach [11]. Tunneled biopsies or EUS-guided biopsies may be needed [8,12]. Bite-on-bite tunneled biopsies may yield diagnostic pathology in about 50% of cases but EUS is preferred. In a 2020 review, EUS demonstrated a sensitivity and specificity of 91.4% and 94.4%, respectively, in detecting T3 cancers. Additionally, this diagnostic modality is preferred because it allows clinicians to assess extraluminal pathology [13].

Age, extent of disease, immunocompromised status, bone marrow involvement, and serum lactate dehydrogenase are all prognostic factors but the exact effect of risk is unknown due to the rarity of the disease. Due to limited data, there is also a wide range of survival, ranging from 26% to 73% [14]. Furthermore, due to the paucity of patients being treated for primary esophageal DLBCL, there is no standard treatment approach for these patients. While chemotherapy, radiation, surgery, and endoscopic resection are optional treatment modalities, many oncologists treat primary esophageal DLBCL similarly to other non-Hodgkin’s lymphoma subtypes [15,16]. The most common chemotherapy regimen is R-CHOP (rituximab, cyclophosphamide, doxorubicin, vincristine sulfate, and prednisone) [17]. This chemotherapy can be used in conjunction with radiation to shrink masses. Surgical subtotal esophagectomy is sometimes considered in localized disease; however, in advanced disease, chemotherapy or radiation is pursued before surgical intervention [14].

## Conclusions

DLBCL is a rare primary esophageal malignancy and few cases have been documented in medical literature. Non-specific symptoms and deep pathologic tissue make this a difficult diagnosis to make. Our case report highlights an uncommon diagnosis and helps bring clinical awareness to this malignancy to help providers pursue further testing when appropriate. Further research into the treatment strategies for primary DLBCL would be beneficial given the rarity of the malignancy.

## References

[1] Liu Y, Barta SK (2019). Diffuse large B-cell lymphoma: 2019 update on diagnosis, risk stratification, and treatment. Am J Hematol.

[2] Ghimire P, Wu GY, Zhu L (2010). Primary esophageal lymphoma in immunocompetent patients: two case reports and literature review. World J Radiol.

[3] Swerdlow SH, Campo E, Pileri SA (2016). The 2016 revision of the World Health Organization classification of lymphoid neoplasms. Blood.

[4] Graus F, Ariño H, Dalmau J (2014). Paraneoplastic neurological syndromes in Hodgkin and non-Hodgkin lymphomas. Blood.

[5] Berman MD, Falchuk KR, Trey C, Gramm HF (1979). Primary histiocytic lymphoma of the esophagus. Dig Dis Sci.

[6] Freeman C, Berg JW, Cutler SJ (1972). Occurrence and prognosis of extranodal lymphomas. Cancer.

[7] Pasqualucci L, Dalla-Favera R (2018). Genetics of diffuse large B-cell lymphoma. Blood.

[8] Qu J, Zhuang Y, Zheng D, Huang F, Zhang S (2021). Primary esophageal lymphoma: clinical experience in diagnosis and treatment. Cureus.

[9] Soon MS, Yen HH, Soon A, Lin OS (2005). Primary esophageal B-cell lymphoma: evaluation by EUS. Gastrointest Endosc.

[10] Dawson IM, Cornes JS, Morson BC (1961). Primary malignant lymphoid tumours of the intestinal tract. Report of 37 cases with a study of factors influencing prognosis. Br J Surg.

[11] Sanchez-Bueno F, Garcia-Marcilla JA, Alonso JD, Acosta J, Carrasco L, Piñero A, Parrilla P (1998). Prognostic factors in primary gastrointestinal non-Hodgkin's lymphoma: a multivariate analysis of 76 cases. Eur J Surg.

[12] Lee DS, Ahn YC, Eom DW, Lee SJ (2016). Primary esophageal mucosa-associated lymphoid tissue lymphoma diagnosed by using stacked forceps biopsy. Dis Esophagus.

[13] Thakkar S, Kaul V (2020). Endoscopic ultrasound stagingof esophageal cancer. Gastroenterol Hepatol (N Y).

[14] Inayat F, Munir A, Wahab A, Younus F, Zafar F, Ullah W (2018). Primary esophageal diffuse large B-cell lymphoma: a comparative review of 15 cases. J Investig Med High Impact Case Rep.

[15] Raderer M, Paul de Boer J (2010). Role of chemotherapy in gastric MALT lymphoma, diffuse large B-cell lymphoma and other lymphomas. Best Pract Res Clin Gastroenterol.

[16] Choi J (2022). Successful endoscopic resection of gastric mucosa-associated lymphoid tissue lymphoma unresponsive to Helicobacter pylori eradication therapy. Clin Endosc.

[17] Kubuschok B, Held G, Pfreundschuh M (2015). Management of diffuse large B-cell lymphoma (DLBCL). Cancer Treat Res.

